# Novel and Stable Dual-Color IL-6 and IL-10 Reporters Derived from RAW 264.7 for Anti-Inflammation Screening of Natural Products

**DOI:** 10.3390/ijms20184620

**Published:** 2019-09-18

**Authors:** Papawee Saiki, Yasuhiro Kawano, Yoshihiro Nakajima, Leo J. L. D. Van Griensven, Koyomi Miyazaki

**Affiliations:** 1 Biomedical Research Institute, National institute of Advance Industrial Science and Technology, Tsukuba, Ibaraki 305-8566, Japan; 2 Health Research Institute, National institute of Advance Industrial Science and Technology, Takamatsu, Kagawa 761-0395, Japan; 3 Plant Research International, Wageningen University and Research Centre, 6708 PB Wageningen, The Netherlands

**Keywords:** IL-6, IL-10, luciferase, anti-inflammation

## Abstract

Our previous study suggested that the interleukin (IL)-6 and IL-10 could serve as good biomarkers for chronic inflammatory disease. We previously established an IL-6 and IL-10 reporters assay that could examine reporter activity along with the reference gene in LPS-induced RAW 264.7 cells. In this study, we described new and stable RAW 264.7 derived dual-color IL-6/gapdh and IL-10/gapdh reporters. This assay allowed us to easily determine relative IL-6 and IL-10 levels with 96-well plate within one step. We evaluated the relative IL-6 and IL-10 levels in the LPS-induced stable cells testing 52 natural products by real-time bioluminescence monitoring and time-point determination using a microplate luminometer. The relative IL-6 and IL-6/IL-10 values decreased by the crude ethanol extracts from nutmeg and by 1′S-1′-acetoxychavicol from greater galangal using real-time bioluminescence monitoring. At the same time, the relative IL-10 was induced. The relative IL-6 and IL-6/IL-10 decreased by crude ethanol extracts from nutmeg and 1′S-1′-acetoxychavicol acetate at 6 h. Only crude ethanol extract from nutmeg induced IL-10 at 6 h. We suggested that the use of these stable cells by real-time monitoring could serve as a screening assay for anti-inflammatory activity and may be used to discover new drugs against chronic inflammatory disease.

## 1. Introduction

Nitric oxide (NO) has a most versatile role in the immune system. It is involved in several diseases such as infectious disease, autoimmune disease, tumors [1], stress-related diseases [2], chronic inflammatory diseases, stroke, diabetes, neurodegenerative disorders [3], and inflammatory bowel disease [4]. Many studies [1,3,4,5] showed that NO is related with pro-inflammatory cytokines interleukin (IL)-6 and anti-inflammatory cytokines IL-10. Lipopolysaccharide (LPS) induces production of NO, IL-6, and IL-10 in macrophage cell lines. IL-10 could inhibit anti-inflammatory IL-6 in activated macrophage cell lines [6]. Recently, balance of pro-inflammatory and anti-inflammatory cytokines has become important for recovery of patients which have inflammatory diseases [7,8,9,10,11,12]. Our previous study also suggested that IL-6/IL-10 balance may restore chronic inflammatory disease, obesity, diabetes type 2 and depression disease [13].

Therefore, we are finding new determination of IL-6 and IL-10 level assay. Luciferase reporter assays are widely used cellular activities, gene expression, intracellular detection of bioactive compound and drug screening both in vitro and vivo [14,15]. The multicolor luciferase reporter assay is suitable for high-throughput analysis of multiple gene expression in vitro assay system. In previous study, we established IL-6 and IL-10 reporters that could determine reporter activity along with gapdh reporter in LPS-induced RAW 264.7 cells. However, it needs transfection of dual IL-6/gapdh and IL-10/gapdh reporters. It is possible that ratio of IL-6:gapdh and IL-6:gapdh will change by each time of transfection. Moreover, it is difficult to use this system with 96-well plate for screening. In this study, we established novel stable RAW 264.7 derived dual-color IL-6/gapdh and IL-10/gapdh reporters. We could determine relative IL-6/gapdh and IL-10/gapdh without transfection. Therefore, we could determine relative IL-6/gapdh and IL-10/gapdh with 96-well plate within one step. It will make determination of IL-6 and IL-10 are less cost and time by these assays. As mentioned above, NO is related with IL-6 and IL-10 levels in macrophage. Therefore, we screened 52 natural products using the inhibition of NO production in RAW 264.7 cells. Then, we screened natural products with these stable cells by real-time bioluminescence monitoring and time-point determination with microplate luminometer. Finally, our assay led to discover a new compound for balancing pro-inflammatory and anti-inflammatory activity

## 2. Results and Discussion

NO plays roles in several physiological and pathological processes which are associated with chronic inflammation [16]. In previous study [5], we succeeded to use screening of NO production inhibition to search for anti-inflammatory compounds. In the present study, we screened various crude extracts for the inhibition of NO production to find anti-inflammation compounds Table 1. Crude extracts were prepared as described in Experimental. Crude extracts at 50, 100, and 150 μg/mL were studied for inhibition of NO production in LPS treated RAW 264.7 cells. The results of NO production inhibition were shown in Figure S1. and showed that crude extracts from turmeric, white pepper, laurel, anise seed, Japanese pepper, liquorice, green pepper, curry leaf, and lemongrass and crude polyphenol extract from *Agaricus b.* and crude polysaccharide extract from *Phellinus l.* at 50, 100, and 150 μg/mL significantly inhibited NO production. The crude extract of greater galangal at 50, 100, and 150 μg/mL was toxic for cells (data not shown), but it showed the strongest inhibition of all extracts at the low concentrations of 5, 10, and 20 μg/mL.

For further analysis the crude extract of greater galangal was fractioned by Wakogel^®^C-300 with Hexane and Acetone and the fractions were determined for inhibition of NO production. The results shown in Figure S2A, the results showed that all fractions strongly inhibited NO production. Fraction 1 was selected and fractioned by HPLC (Figure S3) as described in Experimental. The resulting 6 fractions were again measured for the inhibition of NO production in LPS treated cells. The results showed that HPLC fraction No. 1.3 has the strongest NO production inhibition in Figure S2B. The fraction No. 1.3 was purified by HPLC as before and identified for structure by 1H NMR, 13C NMR, HMQC, HMBC, and COSY. The results from NMR suggested that fraction No. 1.3 is 1′S-1′-Acetoxychavicol acetate. 1H NMR and 13C NMR chemical shifts were compared with published 1H NMR and 13C NMR chemical shifts of 1′S-1′-Acetoxychavicol acetate (ACA) as shown in Table 2

To confirm NO production inhibition of 1′S-1′-Acetoxychavicol acetate, the purified 1′S-1′-Acetoxychavicol acetate at 5, 25, and 100 μM from greater galangal was determined to inhibit NO production. The results showed that 1′S-1′-Acetoxychavicol acetate at 25 and 100 μM are slightly toxic for RAW 264.7 cells (data not shown). 1′S-1′-Acetoxychavicol acetate at 5 μM strongly reduced NO production significantly (Figure S2C).

In previous study, we constructed new IL-6 and IL-10 reporters and developed dual-color luciferase reporter assay for determination of IL-6 and IL-10 promoter expression with relative gene expression of gapdh [17]. In this study, we generated new stable RAW264.7 cell derived dual-color IL-6/gapdh and IL-10/gapdh reporters. The stable RAW 264.7 cells were constructed by transfection of IL-6 or IL-10 and gapdh reporters. After transfection, IL-6/gapdh and IL-10/gapdh transfected RAW 264.7 cells were diluted and treated with Hygromycin B for selection. Several cells were selected and treated at 1 μg/mL LPS. Then, relative IL-6/gapdh IL-10/gapdh levels were determined by real-time bioluminescence monitoring for 48 h. The stable cells which have stable and the strongest IL-6 and IL-10 expression were selected and stocked for further experiments (data not shown).

**Figure 1:**
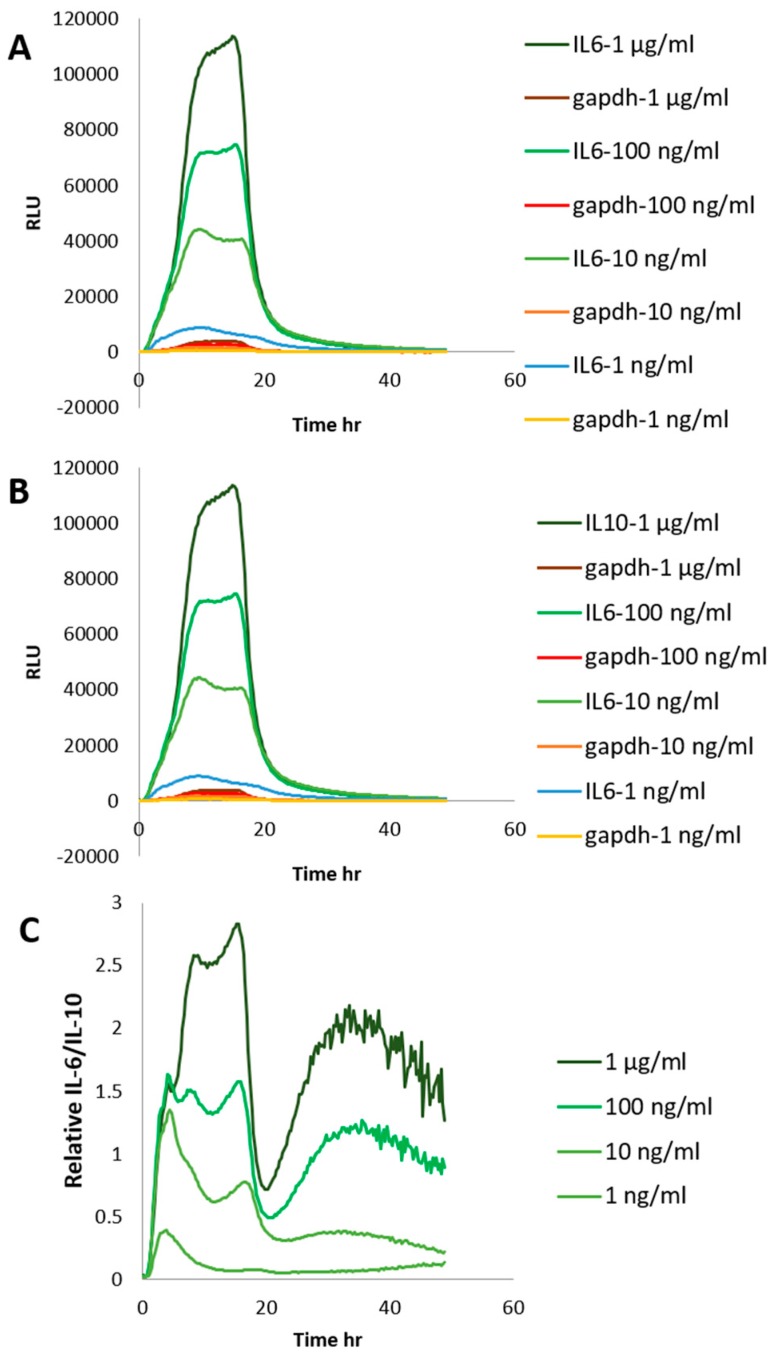
The expression of stable interleukin (IL)-6/gapdh-RAW264.7 cells and IL-10/gapdh-RAW264.7 cells by real-time bioluminescence recording. (**A**): Stable IL-6/gapdh RAW264.7 cells were treated by lipopolysaccharide (LPS) at 1 μg/mL, 100 ng/mL, 10 ng/mL, and 1 ng/mL. (**B**): Stable IL10/gapdh RAW264.7 cells were treated by LPS at 1 μg/mL, 100 ng/mL, 10 ng/mL, and 1 ng/mL. (**C**): Relative IL6/IL10. The results represent the mean of four representative experiments.

To find appropriate concentration of LPS, stable cells were plated in 24-well black plate with clear bottom for 24 h. Cells were replaced with DMEM with 10% FBS, 0.1 mM D-Luciferin potassium salt, 25 mM hepes, and LPS at 1 ng/mL, 10 ng/mL, 100 ng/mL, and 1 μg/mL. Bioluminescence was recorded in real-time with multi-color system under a 5% CO_2_ atmosphere at 37 °C for 48 h. In Figure 1, it appears that LPS response to IL6 and IL10 levels in stable cells are concentration-dependent. LPS also response to relative IL-6/IL-10 are concentration-dependent (Figure 1C).

To evaluate novel stable cells, relative IL-6/gapdh and IL-10/gapdh were measured in LPS-induced stable cells by all extracts as followed in Table 1 with real-time monitoring of dual-color luciferase reporter assay. Stable were plated in 96-well plate for 24 h. The culture medium was replaced with DMEM with 10% FBS, 0.1 mM D-Luciferin potassium salt, 25 mM hepes and samples. After 30 min, cells were treated by LPS at 100 ng/mL. Bioluminescence was recorded in real-time with multi-color system under a 5% CO_2_ atmosphere at 37 °C for 48 h. The results of relative IL-6/gapdh and IL-10/gapdh represent the mean of four representative experiments (data not shown). The results are concluded in Table 1. As mentioned above, we screened all crude extracts for the inhibition of NO production. We found that crude extracts from Anise seed, Japanese pepper and crude polyphenol extract from *Agaricus b.* significantly inhibition NO production, but did not inhibit relative IL-6. However, the results showed that NO production and relative IL-6 were strongly inhibited by crude ethanol extracts from nutmeg, oregano, laurel, long pepper, and greater galangal. The relative IL-10 was stimulated by crude ethanol extract from nutmeg, oregano, laurel, long pepper, and greater galangal. We expected to discover a new compound for balancing pro-inflammatory and anti-inflammatory activity. Therefore, we selected crude extracts that could reduce NO production and IL-6 level, and could induce IL-10 level.

**Figure 2:**
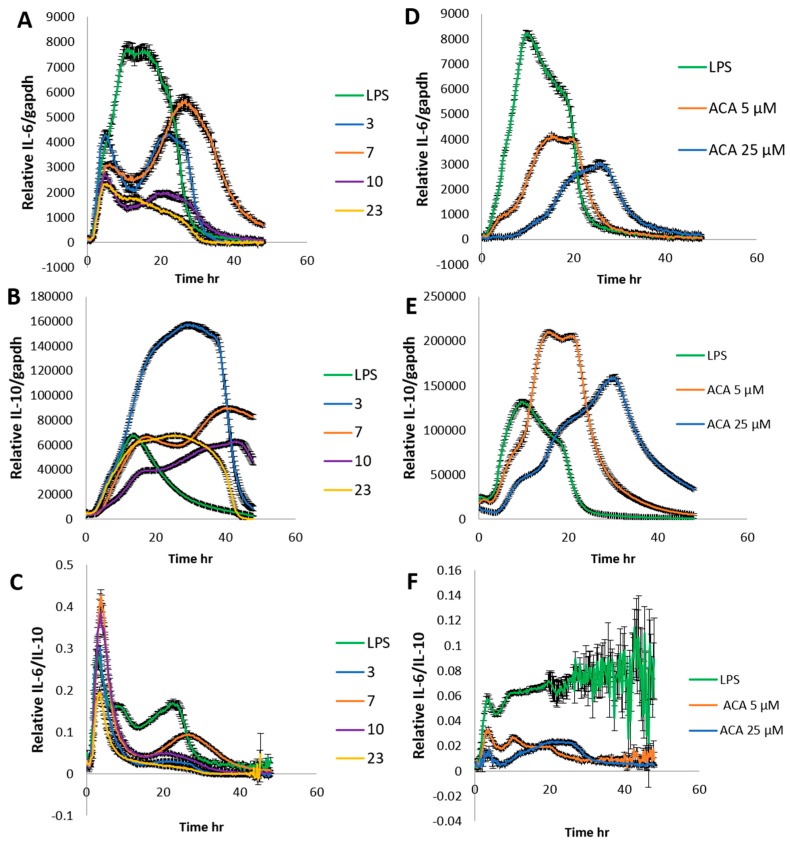
The expression of relative IL-6/gapdh and IL-10/gapdh by real-time bioluminescence recording for 48 h. (**A**) Stable IL-6/gapdh RAW264.7 cells and (**B**) stable IL-10/gapdh RAW264.7 cells were treated with crude ethanol extracts from nutmeg (3), oregano (7), laurel (10), and long pepper (23) at 75 μg/mL and LPS at 100 ng/mL. (**D**) Stable IL-6/gapdh RAW264.7 cells and (**E**) Stable IL-10/gapdh RAW264.7 cells were treated with 1′S-1′-Acetoxychavicol acetate (ACA) at 5, 25 and 100 μM from greater galangal and LPS at 100 ng/mL. (**C**,**F**) Relative IL-6/IL-10. Each value represents the mean ± SD (*n* = 4).

We found that 1′S-1′-Acetoxychavicol acetate from greater galangal strongly inhibited NO production. Therefore, the selected crude extracts and 1′S-1′-Acetoxychavicol acetate were determined relative IL-6 and IL-10 by these stable cells to confirm the results again. The stable cells were plated in 24-well black plate with clear bottom in the DMEM under standard conditions for 24 h. The culture medium was replaced with DMEM with 10% FBS, 0.1 mM D-Luciferin potassium salt, 25 mM hepes and crude ethanol extracts from nutmeg, oregano, laurel and long pepper at 75 μg/mL and 1′S-1′-Acetoxychavicol acetate at 5, 25 and 100 μM from greater galangal. After 30 min, LPS 100 ng/mL was treated into cells. Bioluminescence was recorded in real-time with multi-color system under a 5% CO_2_ atmosphere at 37 °C for 48 h. The results showed in Figure 2. The crude ethanol extracts from nutmeg, oregano, laurel and long pepper at 75 μg/mL and 1′S-1′-Acetoxychavicol acetate at 5 and 25 μM from greater galangal could reduce relative IL-6 and IL-6/IL-10. Whereas, crude ethanol extracts from nutmeg and 1′S-1′-Acetoxychavicol acetate at 5 and 25 μM clearly induced IL-10. 1′S-1′-Acetoxychavicol acetate at 100 μM is toxin for stable cell. Bioluminescence could not be detected (data not shown).

**Figure 3:**
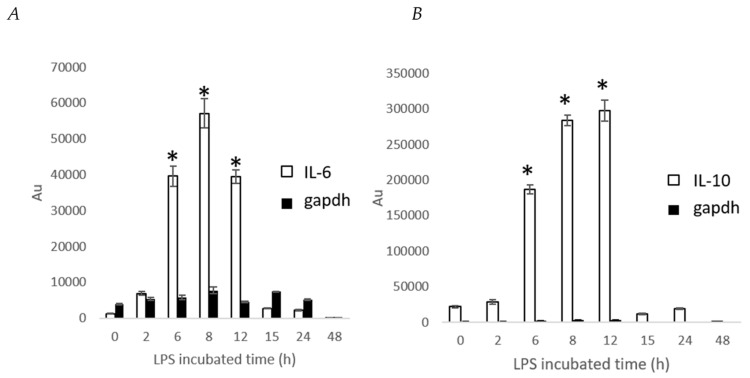
Expression of IL-6 and IL-10 in LPS treated stable cell using a microplate luminometer. Stimulation of stable cells with LPS 100 ng/mL for various time periods. (**A**): IL-6 and gapdh luciferase reporter activity. (**B**): IL-10 and gapdh luciferase reporter activity. Each value represents the mean ± SD (*n* = 4). * *p* < 0.001 versus the LPS group.

In T. Takahashi et al. (2011) [18], stable THP-1–derived IL-8 reporter cell line was determined at time point by microplate luminometer for skin sensitizers screening. Therefore, we also examined stable IL-6 and IL-10 cells with time point by microplate luminometer. First, we stimulated stable cells with LPS 100 ng/mL and determine IL-6 and IL-10 at time point from 0–48 h. The maximum induction of stable IL-6 and IL-10 cells were observed between 6–12 h (Figure 3). The gapdh reporter activity did not significantly change from 0 to 24 h and then gradually decreased at 48 h.

Therefore, we determined the effect of crude ethanol extracts from nutmeg, oregano, laurel, long pepper, and 1′S-1′-Acetoxychavicol acetate on relative IL-6 and IL-10 level at 6 and 8 h by microplate luminometer. Stable cells were plated in 96-well plate for 24 h. The culture medium was replaced with DMEM with 10% FBS and crude ethanol extracts from nutmeg, oregano, laurel, and long pepper at 75 μg/mL and 1′S-1′-Acetoxychavicol acetate at 5, 25, and 100 μM from greater galangal. After 30 min, LPS 100 ng/mL was treated into cells. After incubation for 6 and 8 h, culture medium was replaced with phosphate-buffered saline (PBS) and Tripluc luciferase assay reagent. After shaking, reporter activity was measured by microplate luminometer. In Figure 3, the results showed that crude ethanol extracts from nutmeg, oregano, laurel and long pepper at 75 μg/mL and 1′S-1′-Acetoxychavicol acetate at 5, 25, and 100 μM from greater galangal could reduce relative IL-6 and IL-6/IL-10 at 6 h. Whereas, we found only crude ethanol extracts from nutmeg could induce relative IL-10 at 6 h.

**Figure 4:**
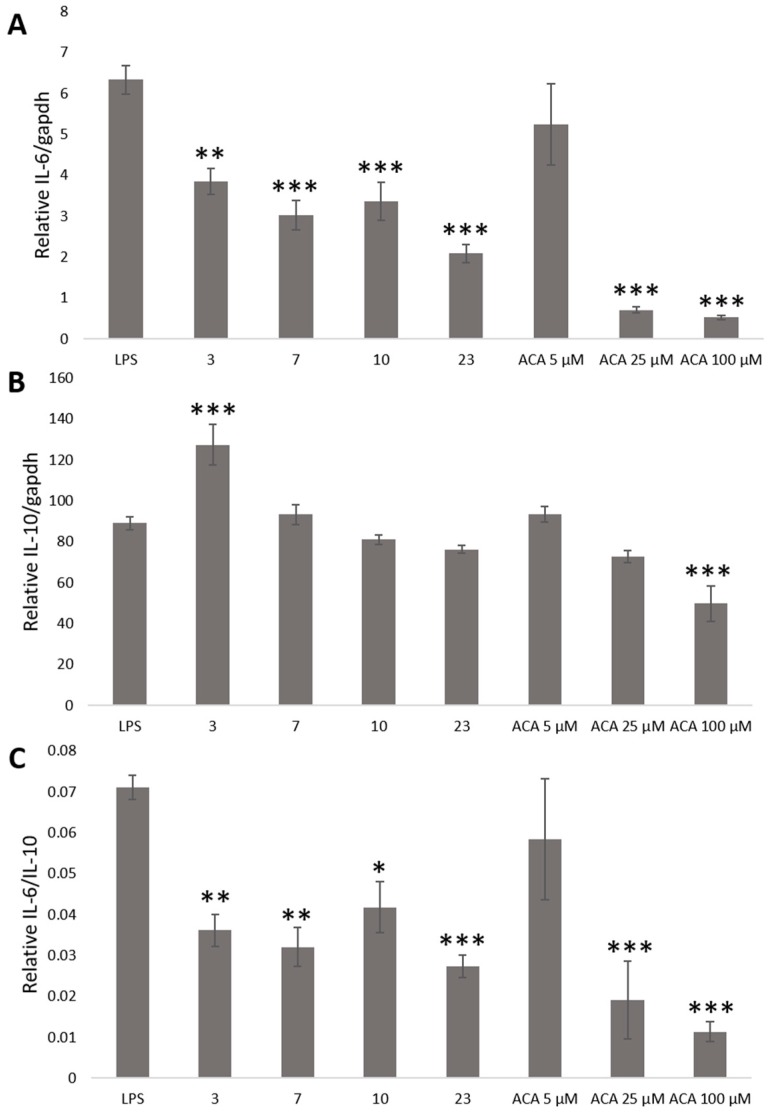
The expression of relative IL-6/gapdh (**A**), IL-10/gapdh (**B**), and IL-6/IL-10 (**C**) by microplate luminometer at 6 h. Stable cells were treated with crude ethanol extracts from nutmeg (3), oregano (7), laurel (10) and long pepper (23) at 75 μg/mL, 1′S-1′-Acetoxychavicol acetate (ACA) at 5, 25, and 100 μM from greater galangal and LPS at 100 ng/mL. Each value represents the mean ± SD (*n* = 4). * *p* < 0.05, **p < 0.01, *** *p* < 0.001 versus the LPS group.

In Figure 4, the results showed that crude ethanol extracts from nutmeg, oregano, laurel, and long pepper at 75 μg/mL and 1′S-1′-Acetoxychavicol acetate at 5, 25, and 100 μM from greater galangal could reduce relative IL-6/IL-10 at 8 h. Crude ethanol extracts from laurel and long pepper at 75 μg/mL and 1′S-1′-Acetoxychavicol acetate at 5, 25 and 100 μM could reduce relative IL-6. Whereas, we found that crude ethanol extracts from nutmeg and oregano at 75 μg/mL could induce relative IL-10 significantly.

These results showed that crude ethanol extracts from nutmeg, oregano, laurel, and long pepper at 75 μg/mL and 1′S-1′-Acetoxychavicol acetate at 5, 25, and 100 μM from greater galangal could reduce relative IL-6/IL-10 at 6 and 8 h. Whereas, reduction of relative IL-6 and induction of IL-10 were changed by 6 and 8 h. The reporter activity from microplate luminometer at time point is different from real-time bioluminescence recording. We could detect clearly that S-1′-Acetoxychavicol acetate at 5 and 25 μM from greater galanga induced relative IL-10 by real-time bioluminescence recording, but we could not detect induction of relative IL-10 by S-1′-Acetoxychavicol acetate at 5 and 25 μM at 6 and 8 h using microplate luminometer. Moreover, we could detect crude ethanol extracts from nutmeg and oregano induced IL-10, but they could not reduce IL-6 at 8 h. We previously reported that crude polysaccharide from *Phellinus l.* significantly inhibited IL-6 at 6 and 24 h and IL-10 level at 24 h using Quantitative reverse transcriptase polymerase chain reaction (RT-PCR) analysis. In this study, we found that Crude polysaccharide from *Phellinus l.* reduced IL-6 level in these stable cells, but did not reduce IL-10 level. Although, T. Takahashi et al. [18] recommended determination of reporter activity from stable cell by microplate luminometer, these results suggested that this method is not suitable for IL-6 and IL-10 screening. It indicates that determination of reporter activity from stable cell by real-time bioluminescence recording is more suitable for screening.

**Figure 5:**
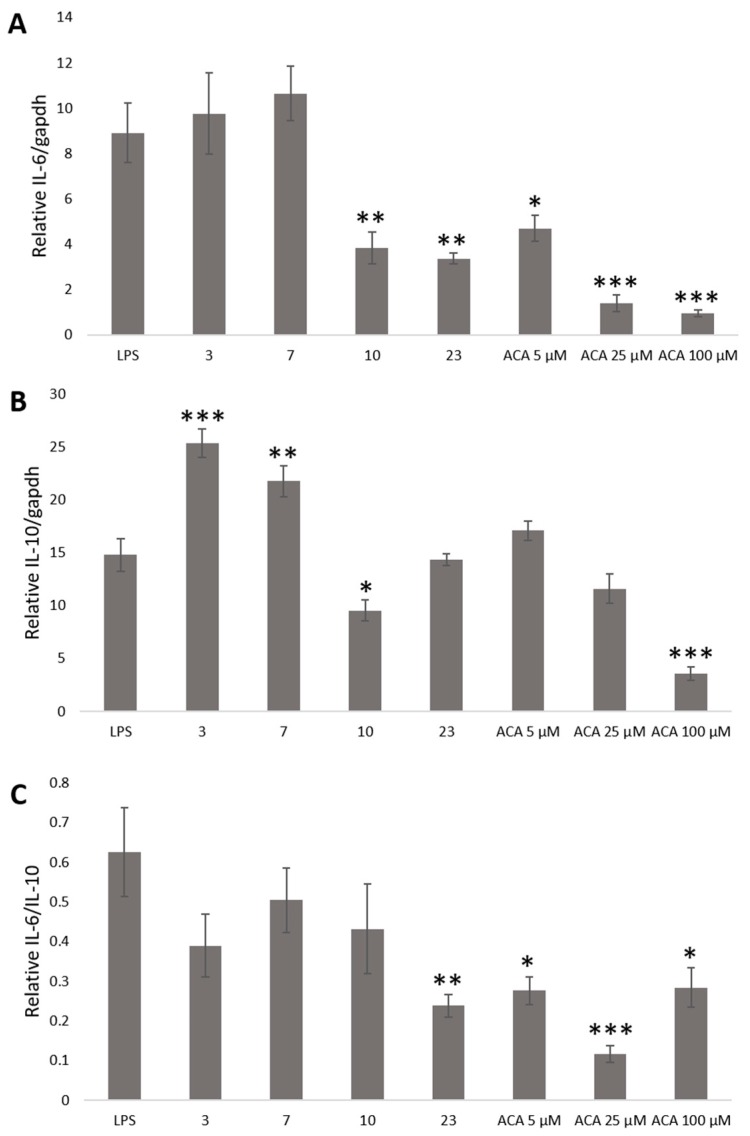
The expression of relative IL-6/gapdh (**A**), IL-10/gapdh (**B**) and IL-6/IL-10 (**C**) by microplate luminometer at 8 h. Stable cells were treated with crude ethanol extracts from nutmeg (3), oregano (7), laurel (10), and long pepper (23) at 75 μg/mL and 1′S-1′-Acetoxychavicol acetate (ACA) at 5, 25, 100 μM from greater galangal and LPS at 100 ng/mL. Each value represents the mean ± SD (*n* = 4). * *p* < 0.05, ** *p* < 0.01, *** *p* < 0.001 versus the LPS group.

Nutmeg (*Myristica fragrans*) has been studied for anti-oxidant [19,20,21,22,23], anti-microbial [24,25], hepatoprotection [26], anti-cancer [27], anti-hyperglycaemia, anti-hyperlipidaemia [28], anti-fungal [29], and anti-inflammation [30,31,32,33]. It was reported that extract of nutmeg reduced NO, IL-6, and IL-10 in LPS-stimulated RAW 264.7 cells by enzyme-linked immunosorbent assay (ELISA) and multiplex bead-based cytokine assay. However, we found that nutmeg extract induced relative IL-10 by real-time bioluminescence monitoring. ELISA and multiplex bead-based cytokine assay are determination of cytokines level at time-point as microplate luminometer assay. Therefore, we could not detect induction of IL-10 level by nutmeg extract. It clearly indicated that our assay has merit, and is more suitable for determination of IL-6 and IL-10 levels Figure 5.

S-1′-Acetoxychavicol acetate from greater galangal (*Alpinia galangal*) has been studied mainly for anti-cancer [34,35,36,37,38,39]. Although, it was reported that S-1′-Acetoxychavicol acetate from *Languas galangal* suppressed NO production in LPS-induced RAW 264.7 cell with dose-dependent [40]. However, we found that S-1′-Acetoxychavicol acetate from greater galangal could reduce IL-6 level and increase IL-10 level. This discovery may lead to develop new drug for balancing pro-inflammatory and anti-inflammatory activity and chronic inflammatory disease.

## 3. Materials and Methods

### 3.1. Cell Culture

The mouse Abelson leukemia virus transformed monocyte macrophage cell line (RAW 264.7 cells) were obtained from Riken Cell Bank (Tsukuba, Japan). RAW 264.7 cells and stable cells were cultured in Dulbecco’s modified Eagle’s medium (DMEM, FUJIFILM Wako Pure Chemical Corporation, Osaka, Japan) containing 10% fetal bovine serum (FBS, Biowest, Tokyo, Japan) at 37 °C in 5% CO_2_ in a humidified incubator.

### 3.2. Extraction, Isolation, and Purification of 1′S-1′-Acetoxychavicol Acetate

Dried Samples (No.1-48) in Table 1 were kindly provided by S&B Foods (Tokyo, Japan). Dried samples were extracted by Ethanol (Wako Pure Chemical Industries, Ltd., Osaka, Japan). Dried fruit body powder of *Agaricus blazei* (*Agaricus b.*), *Ganoderma lucidum* (*Ganoderma l.*), and *Phellinus linteus* (*Phellinus l.*) were extracted using ethanol at 70 °C for 24 h. The solution was filtered to obtain a crude ethanol extract. The tissue residue was then autoclaved with 4 volumes of water at 121 °C for 30 min. After filtration, half the amount of the clear filtrate was added to 2 vol of ethanol and at −20 °C for 4 h. The resulting precipitate was dissolved in water to obtain the crude polysaccharide extract. The remaining half of the clear water solution was evaporated, and the dry residue dissolved in dimethyl sulfoxide (DMSO) for the crude polyphenol extract. All extracts were evaporated and dissolved in ethanol or distilled water for bioassay.

The crude ethanol extract from greater galangal was fractionated by Wakogel^®^C-300 (Wako Pure Chemical Industries, Ltd. (Osaka, Japan) with Hexane:Acetone (4:1) to (1:1) (Wako Pure Chemical Industries, Ltd. (Osaka, Japan) We used the NO inhibitory effect of the different fractions to search for the presence of anti-inflammatory compounds [5]. Isolation and purification of the active materials was done by high performance liquid chromatography (HPLC) (Tosoh Model CCPD computer-controlled pump, Tokyo, Japan) equipped with a Capcell PAK C18 5 μM, 10 mm i.d. × 250 mm column (Shiseido Co., Ltd., Tokyo, Japan), and eluted with gradient 50% methanol with 0.1% formic acid and 100% methanol with 0.1% formic acid at a flow rate of 1.5 mL/min and detected using a Jasco UV wavelength detector at 254 nm (JASCO International Co., Ltd., Tokyo, Japan). After we isolated 1′S-1′-Acetoxychavicol acetate, we purified 1′S-1′-Acetoxychavicol acetate again by HPLC as same as condition again.

### 3.3. Analysis of Nuclear Magnetic Resonance Spectroscopy (NMR)

10 mg of dried 1′S-1′-Acetoxychavicol acetate was exchanged in a Chloroform-D1 0.03 col.% TMS. One-dimensional NMR spectroscopy (1H NMR and 13C NMR) and two-dimensional NMR (COSY, HMBC, and HMQC) were performed using a Bruker 500 MHz NMR (Bruker, Yokohama, Japan).

### 3.4. Nitric Oxide (NO) Production

RAW 264.7 cells were cultured in DMEM containing 10% FBS at 37 °C in 5% CO_2_ in a humidified incubator. RAW 264.7 cells (8 × 10^5^ cells per mL) 200 μL were plated in 96-well plate in the DMEM under standard conditions for 24 h. Then, RAW 264.7 cells were treated with samples in Table 1 at 50, 100, and 150 μg/mL and LPS at 1.25 μg/mL [5]. After 15 h, the activated cells’ culture medium was transferred to a new 96-well plate. Griess reagent (Sigma-Aldrich Co., St. Louis, MO, USA) was added and incubated at 37 °C for 15–20 min. The absorbance was read at 540 nm on an iMarkTM Microplate Reader (Bio-Rad Laboratories, Inc., Berkeley, CA, USA). The percentage of NO production was calculated and compared with that of LPS treated cells.

### 3.5. Cell Proliferation Assay

Cell proliferation assay is a colorimetric method for determining the mitochondrial enzyme activity which reflects the number of viable cells. RAW 264.7 cells or stable RAW 264.7 cells (8 × 105 cells per·mL) 200 μL were plated in 96-well plate in DMEM under the standard conditions for 24 h. Then, RAW 264.7 cells were treated with samples and LPS (1.25 μg/mL). After 15 h, the cell culture medium was drained. CellTiter 96^®^ AQueous One Solution Reagent (Promega Corp., Madison, WI, USA) was added to cells and incubated at 37 °C for 20–30 min. The solution was transferred to a new 96-well plate. Its absorbance was read at 490 nm on an iMarkTM Microplate Reader. The absorption due to the proliferating cells was determined and compared to that of untreated RAW 264.7 cells.

### 3.6. Generation of Stable RAW 264.7 Cell Lines

RAW 264.7 cells (7 × 10^5^ cells/mL) were treated in 35-mm dish with 2 mL cells for 24 h. IL-6 or IL-10 reporter plasmid [17] and Gapdh-SLR reporter plasmid [18] were co-transfected by Hilymax transfection reagent (Dojindo Molecular Technologies, Inc., Rockville, MD, USA) according to the manufacturer’s instructions for 24 h. Then, transfected cells were diluted and culture into 10 cm culture dishes with Hygromycin B at 200μg/mL (Invitrogen, Tokyo, Japan) for cell selection. Several selected cells (7 × 10^5^ cells/mL) were transferred into 35-mm dish with 2 mL cells for 24 h. The culture medium was replaced with DMEM with 10% FBS, 0.1 mM D-Luciferin potassium salt (Wako Pure Chemical Industries, Ltd., Osaka, Japan), 25 mM hepes (Thermo Fisher Scientific Inc., Waltham, MA, USA) and 1 μg/mL LPS from Escherichia coli O26:B6 (Sigma-Aldrich Corporation, St. Louis, MI, USA). For evaluation, bioluminescence was recorded in real-time for 1 min in the presence or absence of optical filter (R60-long pass filter, HOYA, Tokyo, Japan) at 19-min intervals under a 5% CO_2_ atmosphere at 37 °C for 48 h using an AB-2500 Kronos (ATTO, Tokyo, Japan) as described previously [41].

### 3.7. Determination IL-6 and IL-10 Level by Stable Cells Using a Bioluminescence Measurement System for Living Cells by Real-Time Monitoring

Stable RAW 264.7 cells (6 × 10^5^ cells per mL) 500 μL were plated in 24-well black plate with clear bottom (Wallac Oy, Turku, Finland) in the DMEM under standard conditions for 24 h. Cells were replaced with DMEM with 10% FBS, 0.1 mM D-Luciferin potassium salt, 25 mM hepes and samples. After 30 min, LPS at 1 ng/mL, 10 ng/mL, 100 ng/mL and 1 μg/mL were added to the cells. Bioluminescence was recorded in real-time for 10 s in the presence or absence of theR60-long pass filter, HOYA, Tokyo, Japan) under a 5% CO_2_ atmosphere at 37 °C for 48 h using a WSL-1565 Kronos HT (ATTO, Tokyo, Japan).

For screening, stable RAW 264.7 cells (8 × 10^5^ cells per mL) 200 μL were plated in 96-well black plate with clear bottom (Thermo Fisher Scientific, Waltham, MA, USA) in the DMEM under standard conditions for 24 h. The culture medium was replaced with DMEM with 10% FBS, 0.1 mM D-Luciferin potassium salt, 25 mM hepes and extracts at 25, 50 and 75 μg/mL. After 30min, LPS at 100 ng/mL was added to the cells. Bioluminescence was recorded in real-time for 10 s in the presence or absence of the R60-long pass filter, HOYA, Tokyo, Japan) under a 5% CO_2_ atmosphere at 37 °C for 48 h using a WSL-1565 Kronos HT.

### 3.8. Determination IL-6 and IL-10 Level by Stable Cells Using a Microplate Luminometer

Stable RAW 264.7 cells (8 × 10^5^ cells per mL) 200 μL were plated in 96-well plate with black frame and transparent blank wells (PerkinElmer, Waltham, MA, USA) in the DMEM under standard conditions for 24 h. Cells were replaced with DMEM with 10% FBS and samples. After 30 min, LPS at 100 ng/mL was treated into cells. After 6 and 8 h incubation, cells were replaced with 1:1 sterile phosphate-buffered saline (PBS) and Tripluc luciferase assay reagent (TOYOBO Co., Ltd., Osaka, Japan). Then, shake the plate for 10 min at room temperature. Luciferase activity was determined for 10 s in the presence or absence of optical filter (R60-long pass filter, HOYA, Tokyo, Japan) using a microplate luminometer with a multi-color detection system, Phelios AB-2350 (Atto Co., Tokyo, Japan)

### 3.9. Statistical Analysis

Statistical analyses were performed by one-way analysis of variance (ANOVA) with Dunnett’s test for selected pairs. The statistical software “EZR” was used for statistical analysis [42]. Values are indicated as means ± SE. The significant differences are shown as probability values.

## 4. Conclusions

We generated new stable RAW 264.7 derived IL-6/gapdh and IL-10/gapdh reporters cell lines. We evaluated these stable cells with LPS and found that LPS response to IL-6 and IL-10 levels in stable cells are concentration-dependent. Our previous study [5] suggested that inhibition of NO production is related with inhibition of IL-6 in RAW 264.7 cells. First, we screened ethanol crude extracts for inhibition of NO production. We found that crude extract from greater galangal has the strongest of NO production inhibition. Then, we isolated, purified and identified S-1′-Acetoxychavicol acetate from greater galangal. We found that S-1′-Acetoxychavicol acetate from greater galangal dose dependently inhibited NO production. Then, we screened ethanol crude extracts for relative IL-6 and IL-10 by these stable cells with real-time bioluminescence monitoring. We found that ethanol crude extracts from nutmeg and S-1′-Acetoxychavicol acetate from greater galangal could reduce relative IL-6 and relative IL-6/IL-10. They also could induce relative IL-10. We also determined IL-6 and IL-10 level at time point by microplate luminometer. It suggests that this method is not suitable for screening IL-6 and IL-10 by these stable cells. Our observations suggest that stable RAW 264.7 derived IL-6 and IL-10 reporters cell lines could serve to perform relative IL-6 and IL-10 screening by real-time bioluminescence monitoring. S-1′-Acetoxychavicol acetate from greater galangal could balance pro-inflammatory IL-6 and anti-inflammatory IL-10 cytokines that may contribute to the prevention of chronic inflammatory disease.

## Figures and Tables

**Table 1:** The summary of results from anti-inflammation screening assays.

		NO Production			Relative IL-6/Gapdh			Relative IL-10/Gapdh		
		Extract Conc. (μg/mL)			Extract Conc. (μg/mL)			Extract Conc. (μg/mL)		
No.	Sample Name	150	100	50	75	50	25	75	50	25
1	cinnamon	0	0					●	●	●
2	turmeric	0	0	0	0	0	0	0	0	
3	nutmeg	0	0		0	0	0	●	●	●
4	black pepper	0	0		0	0	0	0	0	
5	white pepper	0	0	0	0	0	0	0	0	
6	red pepper							●	●	●
7	oregano	0			0			●	●	
8	basil							●		
9	parsley				0			●	●	●
10	laurel	0	0	0	0	0	0	●	●	●
11	cardamom	0						●		
12	cumin			0				●	●	
13	cilantro				0			●	●	●
14	fennel				0	0	0	●	●	●
15	anise seed	0	0	0				●	●	●
16	allspice							●	●	●
17	caraway							●	●	●
18	clove				0					
19	Japanese pepper	0	0	0						
20	ginger	0						●	●	●
21	star anise							●		
22	celery seed							●	●	●
23	long Pepper	0	0		0	0	0	●	●	●
24	fenogreek							●	●	●
25	mace				0	0	0	●	●	●
26	dil Seed							●	●	●
27	kaffir lime leaf				0	0	0	●	●	●
28	summer savory							●	●	
29	kaba				0	0		●	●	●
30	sage				0	0				
31	thyme							●	●	●
32	taragon				0			●	●	●
33	marjoram							●	●	●
34	rosemary	0	0		0	0				
35	Chinese pepper	0			0	0	0			
36	liquorice	0	0	0	0	0	0	0	0	0
37	garlic							●	●	●
38	green Pepper	0	0	0	0	0	0	0		
39	paprika							●	●	●
40	horse radish							●	●	●
41	mustard							●	●	●
42	curry leaf	0	0	0	0	0	0	0		
43	spearmint									
44	dillweed									
45	peppermint	0	0							
46	lemongrass	0	0	0	0	0				
47	Saffron									
	extract conc. (μg/mL)	20	10	5						
48	greater galangal	0	0	0	0	0	0	0	0	●
	extract conc. (μg/mL)	150	100	50						
49	crude polysaccharide extract from *Agaricus b.*							●	●	●
50	crude polyphenol extract. from *Agaricus b*	0	0	0				●	●	●
51	crude polysaccharide extract from *Ganoderma l.*							●	●	●
52	crude polysaccharide extract from *Phellinus l.*	0	0	0	0	0				
	0	decreased								
	●	increased								

**Table 2:** The 1H NMR and 13C NMR chemical shifts of 1′S-1′-Acetoxychavicol acetate (ACA) and fraction No. 1.3 (δ in ppm).

	ACA	1.3	ACA	1.3
	A. Khalijah, et al.		H. Azuma et al.	
Carbon Number	13C (ppm)	13C (ppm)	1H (ppm)	1H (ppm)
1	150.5, s	150.6, s		
OCOCH_3_	169.4, s	169.4, s		
OCOCH_3_	21.2, q	21.2, q	2.29 (s, 3H)	2.25 (s, 3H)
2	121.7, d	121.8, d	7.08 (d, 8.5, 1H)	7.07 (d, 8.5, 1H)
3	128.5, d	128.6, d	7.37 (d, 8.5, 1H)	7.36 (d, 8.5, 1H)
4	136.5, s	136.6, s		
5	128.5, d	128.6, d	7.37 (d, 8.5, 1H)	7.36 (d, 8.5, 1H)
6	121.7, d	121.8, d	7.08 (d, 8.5 1H)	7.07 (d, 8.5, 1H)
1′	75.6, d	75.6, d	6.26 (d, 5.9, 1H)	6.26 (d, 5.9, 1H)
OCOCH_3_	169.7, s	169.8, s		
OCOCH_3_	21.3, q	21.3, q	2.10 (s, 3H)	2.10 (s, 3H)
2′	136.1, d	136.2, d	5.98 (ddd, 17.1, 10.5, 5.9, 1H)	5.98 (ddd, 17.1, 10.7, 6.1, 1H)
3′	117.2, t	117.2, t	5.30 (d, 17.1, 1H), 5.25 (d, 10.5, 1H)	5.29 (d, 17.1, 1H), 5.23 (d, 10.5, 1H)

## Supplementary Materials

Supplementary materials can be found at https://www.mdpi.com/1422-0067/20/18/4620/s1.
