# Supplementary material for: Novel and Stable Dual-Color IL-6 and IL-10 Reporters Derived from RAW 264.7 for Anti-Inflammation Screening of Natural Products
Source: Int J Mol Sci. 2019 Sep 18;20(18):4620. doi: 10.3390/ijms20184620 (PMC6769898; doi:10.3390/ijms20184620)
Supplement: Supplementary file 1 [file ijms-20-04620-s001.pdf]

Supplementary Information

The Novel Stable RAW 264.7 Derived Dual-Colour IL-6 and IL-10 Reporters for Anti-Inflammation Screening of Natural Products

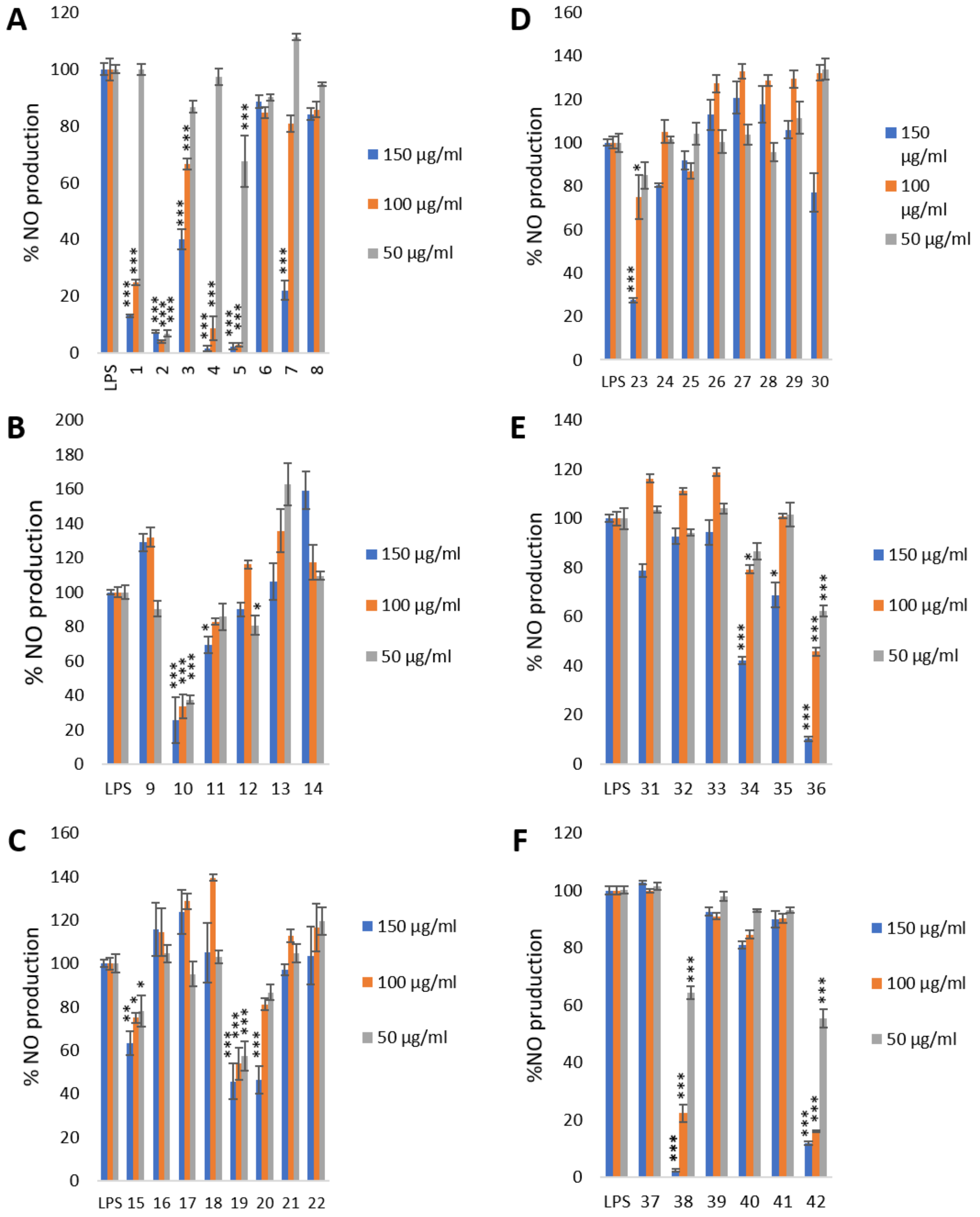

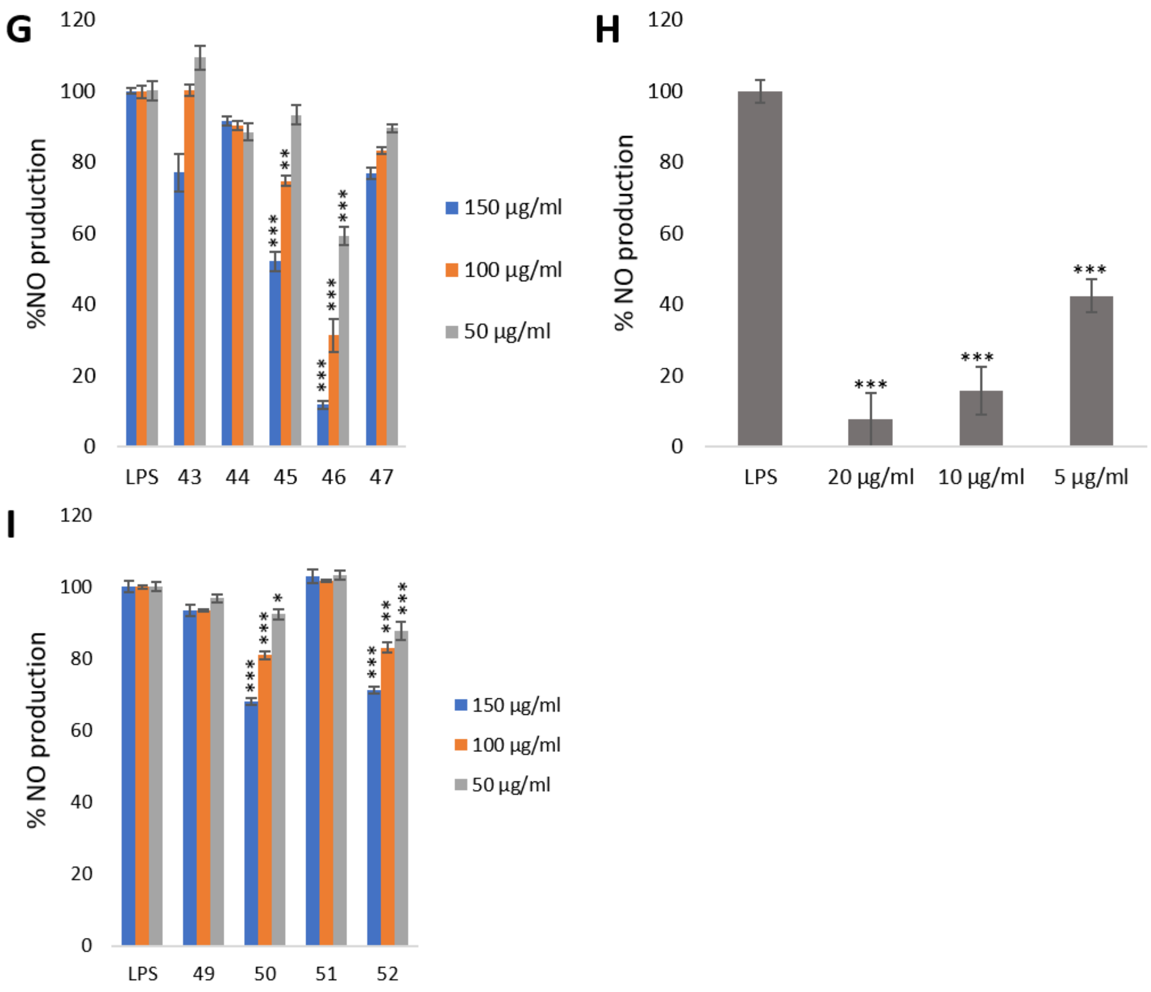

Figure S1. The effect of crude extracts (Table 1) on the inhibition of NO production in LPS treated RAW 264.7 cells. H: Greater Galangal (48) Statistical analyses were performed by means of ANOVA followed by Dunnett's test. Each value represents the mean ± SD ($n = 4$). * $p < 0.05$, ** $p < 0.01$, *** $p < 0.001$ versus the LPS group.

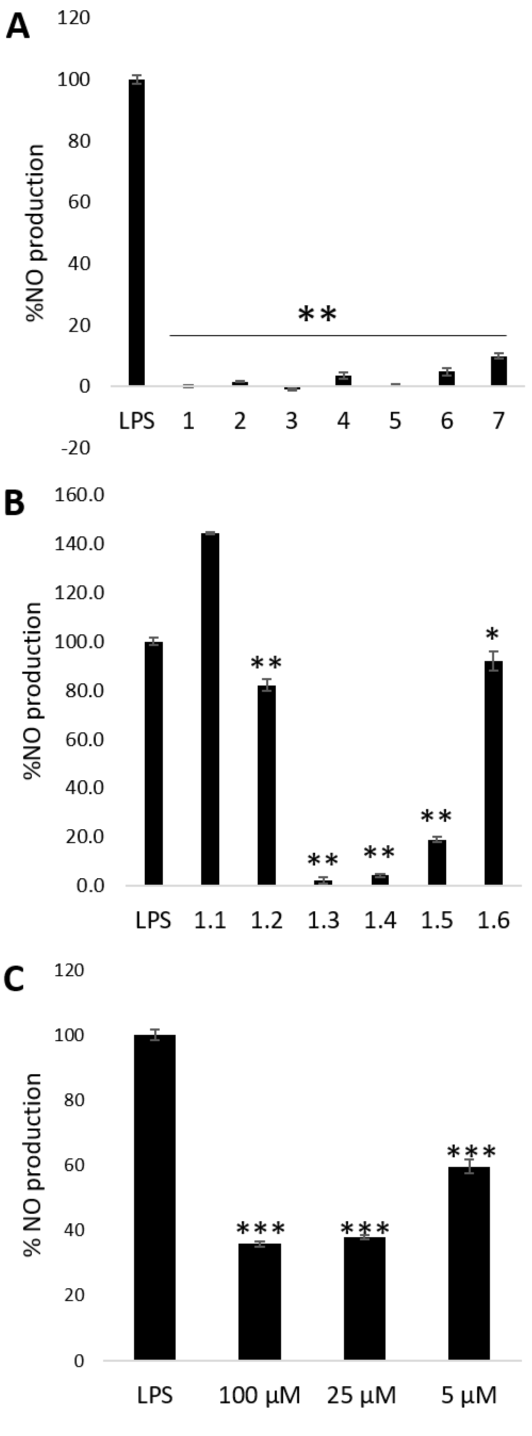

Figure S2. The effect of fractions from Greater Galangal extract on the inhibition of NO production in LPS treated RAW 264.7 cells. **A**: fraction from crude extract **B**: fraction from HPLC **C**: Statistical analyses were performed by means of ANOVA followed by Dunnett's test. Each value represents the mean ± SD (n = 4). * $p < 0.05$, ** $p < 0.001$ versus the LPS group.

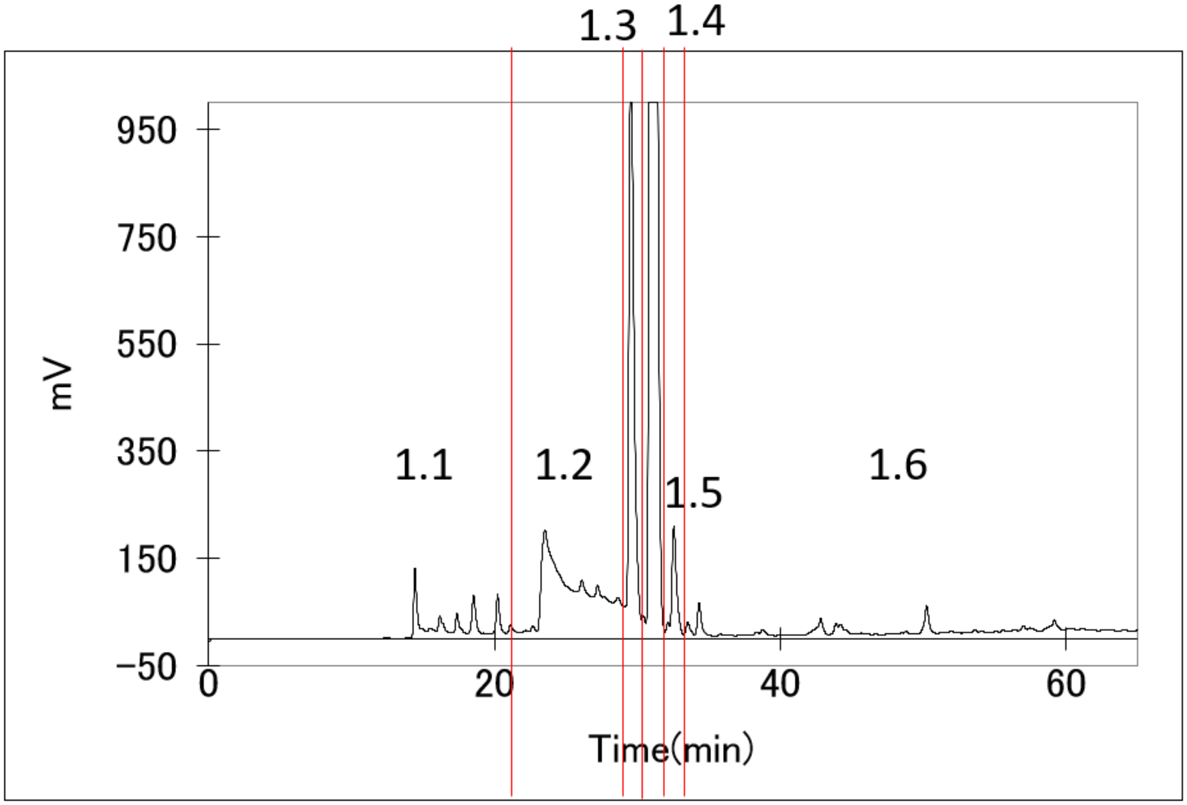

Figure S3. The fractions of crude extract from Greater Galangal by HPLC.

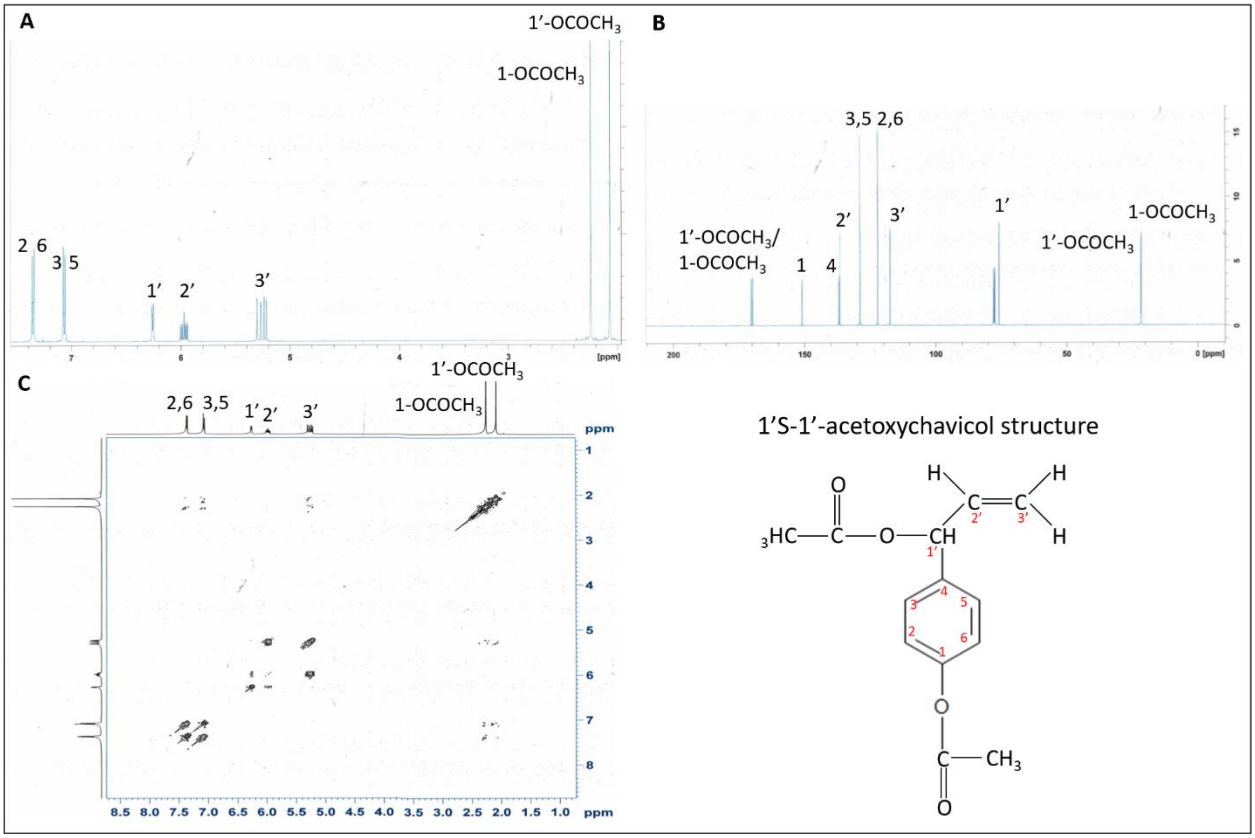

Figure S4. The one-dimensional 1H and 13C and two-dimensional COSY spectra of linoleic acid from (**A**, **B** and **C** respectively).
